# Factors associated with damages and repair costs of reusable flexible bronchoscopes in pediatrics in China

**DOI:** 10.1186/s12887-024-04705-2

**Published:** 2024-04-01

**Authors:** Ruixue Hu, Yanhua Chen, Sixin Jiang, Ting Hu, Juanli Huang, Lu Zhang, Wei Pan, Liangying Yi, Yan Huang

**Affiliations:** 1 Department of Sterile Processing Nursing, West China Second University Hospital, Sichuan University, Chengdu, Sichuan China; 2 Key Laboratory of Birth Defects and Related Diseases of Women and Children (Sichuan University), Ministry of Education, Chengdu, Sichuan China; 3 Operation Management Department, West China Second University Hospital, Sichuan University, Chengdu, Sichuan China; 4 Department of Nursing, West China Second University Hospital, Sichuan University, Chengdu, Sichuan China

**Keywords:** Pediatric flexible bronchoscope, Central reprocessing, Common failure, Cause analysis

## Abstract

**Background:**

Frequent repairs of pediatric flexible bronchoscopes can lead to a huge financial burden for the hospital. This study aimed to investigate the common causes of the failures in pediatric flexible bronchoscopes and propose the measures to prevent the failures.

**Methods:**

This was a retrospective study. We collected repair information of the pediatric flexible bronchoscopes reprocessed in the Department of Sterile Processing at a hospital between September 1, 2018 and September 1, 2022 in order to investigate the causes and possible factors associated with the failures in pediatric flexible bronchoscopes.

**Results:**

The Department of Sterile Processing staff reprocessed the pediatric flexible bronchoscopes 4280 times. A total of 29 failures were identified. The failure rate was 0.678%. The average repair cost was USD7246.60. The common failures in the pediatric flexible bronchoscopes included dim video image, black dots, improper video image display or no image during angulation adjustment, and pressure marks in the insertion tube. The failure rates in flexible electronic bronchoscopes and small-diameter flexible bronchoscopes were 65.5% and 93.1%, respectively. The failure rate in the pediatric flexible bronchoscopes reprocessed by the staff members with less work experience was 75.9%.

**Conclusion:**

The failure rate in the pediatric flexible bronchoscopes was not high but the repair costs were extremely high. The types and size of the flexible bronchoscopes and work experience of the staff members responsible for bronchoscope reprocessing were the possible factors associated with the failure rate in the pediatric flexible bronchoscopes. It is advisable to further optimize the central workflow and management mode for reprocessing the pediatric flexible bronchoscopes, thereby extending their useful life and reducing costs.

## Background

Ikeda, a Japanese physician, was the first to propose the use of flexible bronchoscopes in 1968. Under the promotion of Wood and Fink, flexible bronchoscopy has developed rapidly in pediatrics [1, 2]. Since its initial use more than 40 years ago, the utility of flexible bronchoscopy in the diagnosis and treatment of infectious pulmonary diseases and the removal of foreign bodies in the airways in children has been confirmed [3–5]. Pediatric flexible bronchoscope is an important tool for the diagnosis and treatment of pediatric respiratory diseases.

The flexible bronchoscope is particularly vulnerable to damage in use, cleaning and disinfection due to its special material properties and precise structure. In contrast to adult flexible bronchoscopes, pediatric flexible bronchoscopes have a shorter diameter and more precise structure and are more likely to suffer damage. The damaged flexible bronchoscopes will affect clinical use. Moreover the biofilm may form at the site of damage, resulting in infections that are spread via the flexible endoscope [6]. Previous studies have revealed that the infections transmitted by endoscopes after being thoroughly cleaned and disinfected are caused by the biofilm that was present at the site of damage in the endoscope [7]. Furthermore, the damaged flexible endoscope requires high repair costs, leading to an increase in cost per use [8, 9]. The average repair cost per episode of flexible endoscope failure is reported to be as high as USD2959.44 [10]. It is obvious that the hospital may face a huge financial burden as a result of the frequent repairs of the flexible bronchoscope.

The existing studies on flexible bronchoscope repair and failures place a great emphasis on adult flexible bronchoscopes [11], however, the studies on pediatric flexible bronchoscopes are rare. This study aimed to analyze the failures of pediatric flexible bronchoscopes, identify the potential risk factors for the failures, and propose improvement measures, in order to increase the safety of using pediatric flexible bronchoscopes, extend their useful lives, reduce costs, and ensure patient safety.

## Methods

### Ethics approval

All research methods were carried out in accordance with the relevant guidelines and regulations. This study was conducted in accordance with the Declaration of Helsinki and was approved by the Medical Ethics Committee of West China Second University Hospital, Sichuan University [2021 Medical Scientific Research for Ethical Approval No. (151)].

### General information

There were 11 pediatric flexible bronchoscopes in our hospital with numbers from 1 to 11 marked on each of them. Bronchoscopes 1 and 4 were Olympus BF-P260F fiber bronchoscopes; Bronchoscopes 2 and 3 were Olympus BF-XP260F fiber bronchoscopes; Bronchoscopes 5, 6, 9, 10, and 11 were Olympus BF-XP290 electronic bronchoscopes; Bronchoscopes7 and 8 were Olympus BF-P290 and Olympus BF-Q290 electronic bronchoscopes, respectively. Bronchoscopes 1, 4, 7, and 8 were large-diameter ones, and the rest were small-diameter ones. One or two flexible bronchoscopes might be used for diagnosis or treatment according to the pediatric patient’s age and stage of illness. Bronchoscopes 1 to 4 were purchased in 2013; Bronchoscopes 5 to 8 were purchased in September 2018; and Bronchoscopes 9 to 11 were purchased in November 2019.

All pediatric flexible bronchoscopes in this study had gone through the same cleaning as well as disinfection procedures prior to and after use, which includes leak test, brushing, immersion in enzyme detergent solution, ultrasonic cleaning (1st rinsing - disinfection − 2nd rinsing − 3rd rinsing - air drying - drying with alcohol), manual drying, and packaging. The flexible bronchoscopes with failures were returned to the manufacture for repair.

### Study tools

This was a retrospective case control study. For the purpose of investigating the types and cause of the failures, we collected the information concerning use, cleaning, disinfection, failure, and repair of the pediatric flexible bronchoscopes reprocessed in the Department of Sterile Processing at our hospital between September 1, 2018 and September 1, 2022. The pediatric flexible bronchoscopes with failures were classified into the case group. The samples in the control group were selected in a proportion of 1:4 from the pediatric flexible bronchoscopes without failures. We analyzed the potential contributing factors of failures in the pediatric flexible bronchoscopes.

The use of the pediatric flexible bronchoscopes was checked in our hospital’s health information system. Information regarding cleaning and disinfection was obtained from the tracing system of the Department of Sterile Processing at our hospital. Failure repair information was obtained from the manufacturer. All the flexible bronchoscopes in this study were acquired from the Department of Pediatric Respiratory and Immunology at our hospital. The flexible bronchoscopes were primarily employed in bronohoalveolarlavage, bronchial foreign body removal, and regular bronchoscopy.

### Statistical methods

SPSS21.0 was used for data analysis. Descriptive statistics was used to describe the flexible bronchoscope damages. A chi-square (X^2^) test was used for comparative analysis. The potential factors associated with the failures in pediatric flexible bronchoscopes were investigated using logistic regression analysis. A statistically significant difference was identified by *P* < 0.05.

## Results

**Fig. 1:**
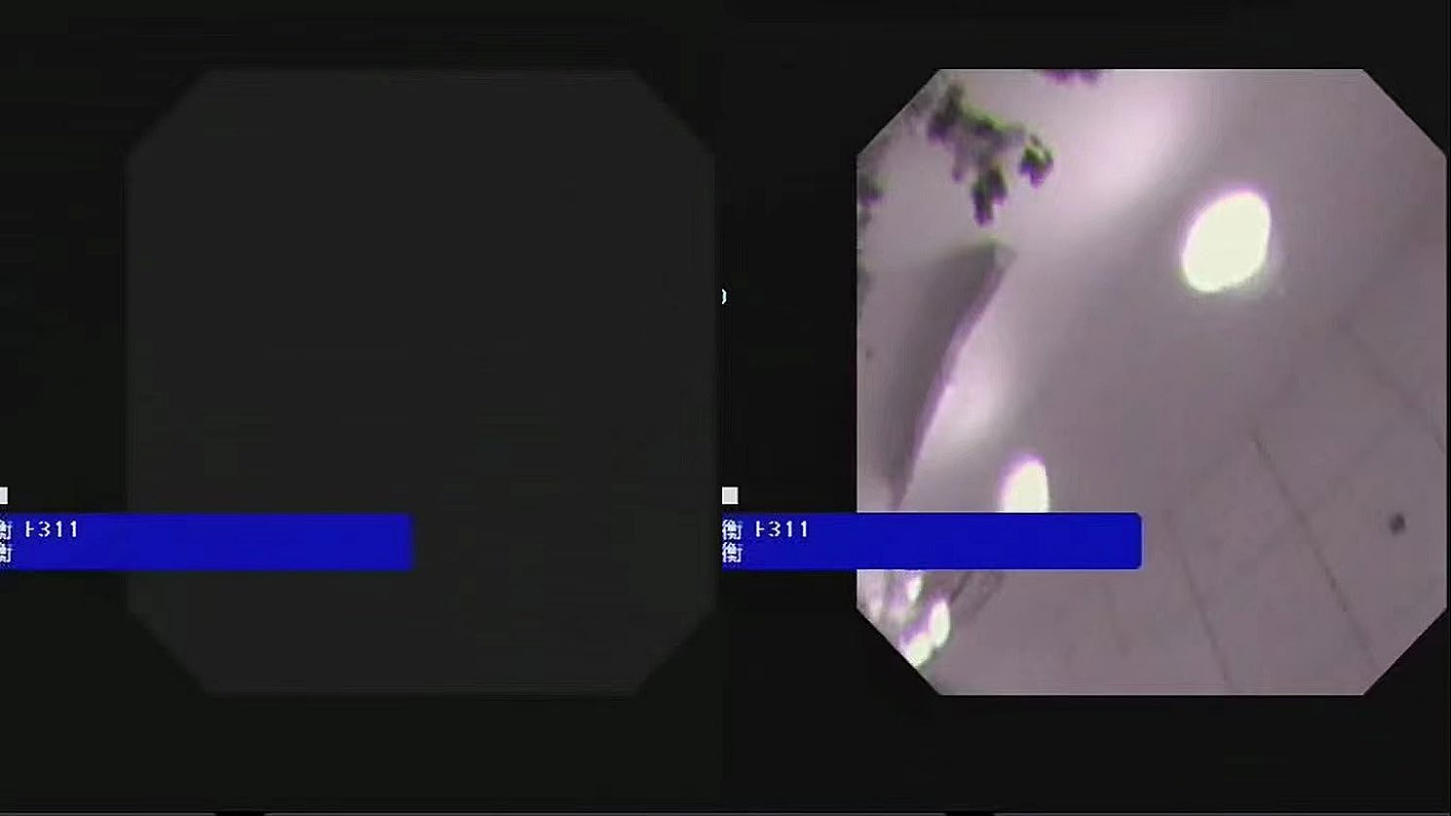
No video image during angulation (left: no video image; right: image was being displayed)

**Fig. 2:**
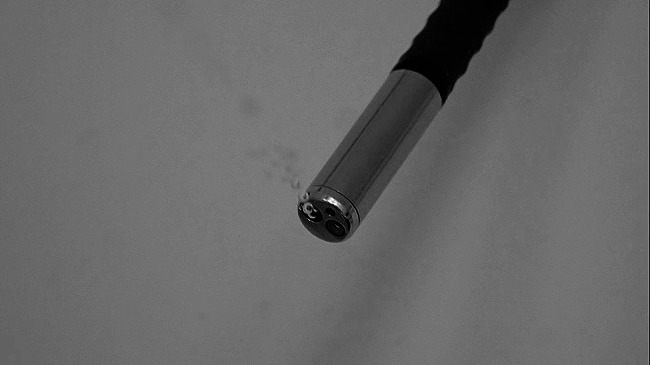
Water leaks in the forceps channel

**Fig. 3:**
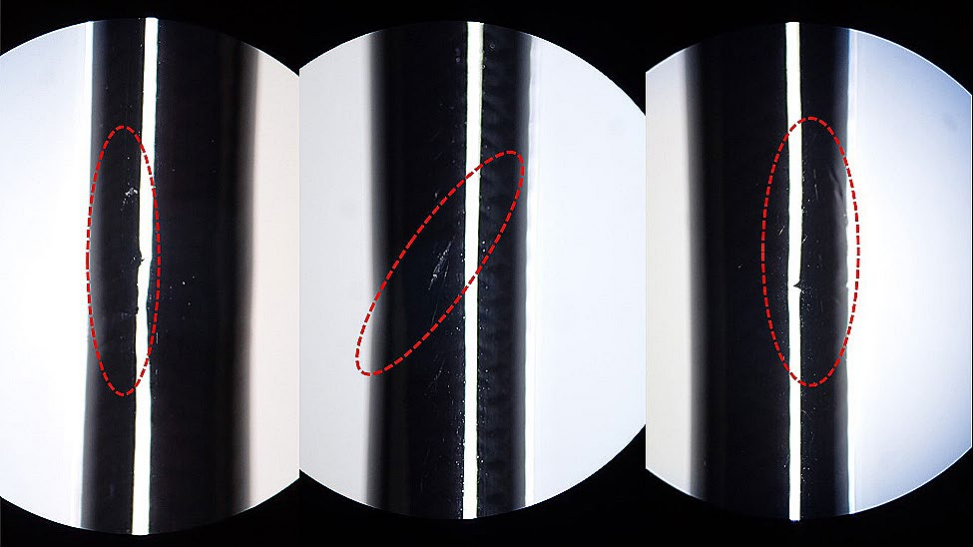
Scratches on the surface of the forceps channel

**Fig. 4:**
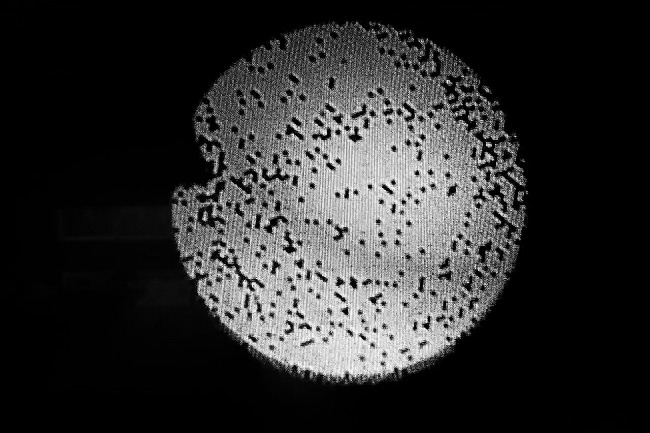
Black dots in the video image

**Fig. 5:**
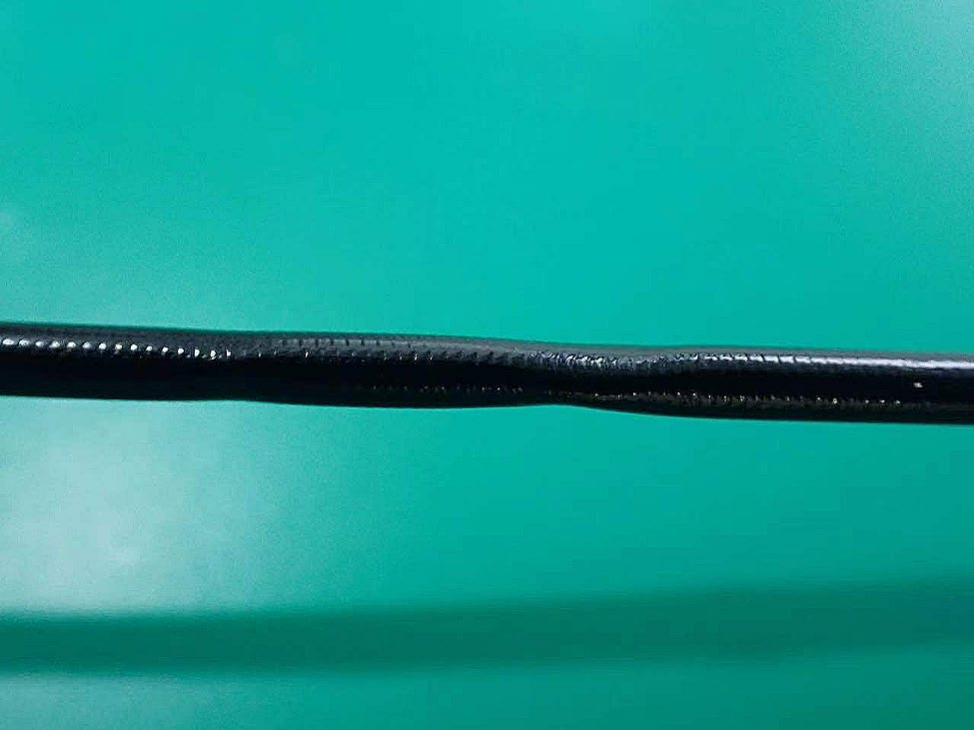
Visible pressure marks in the insertion section

All of the used pediatric flexible bronchoscopes have been delivered to the Department of Sterile Processing immediately after use since September 2018. The pediatric flexible bronchoscopes have been used 4280 times as of September 1, 2022. A total of 29 failures were identified. The failure rate was 0.678%. The average repair cost was USD7246.60. Failure types are shown in Table 1. Typical cases of failures are illustrated in Figs. 1, 2, 3, 4 and 5.

**Table 1:** Types of failures in pediatric flexible bronchoscopes

Description of failure	Solution	Classification of repair	Repair cost (USD)
Water leaks at the bending rubber, many black dots in the video image, and pressure marks or peeling in the insertion tube	Replaced the components of the insertion section	Level A	8142
No upper deflection capability, many black dots in the video image, and connector was oxidized	Replaced the components of the insertion section, and cleaned the connector	Level A	8142
Angle wire was broken, and pressure marks in the insertion tube	Replaced the components of the insertion section	Level A	8142
Water leaks in light guide tube, pressure marks in the insertion tube, no upper deflection capability, and worn-out button	Replaced the components of the insertion section, light guide tube, and button	Level A	8142
The upper deflection control wire was broken, and black dots in the video image	Replaced the components of the insertion section	Level A	8142
No deflection capability, pressure marks in the insertion tube, 6 black dots in the video image, and cracked image	Replaced the components of the insertion section	Level A	8142
No upper deflection capability, and pressure marks in the insertion tube	Replaced the components of the insertion section	Level A	8142
The bending tube did not have a high bending strength, pressure marks in the insertion tube, and around 20 black dots in the video image	Replaced the components of the insertion section	Level A	8142
Dim video image, and pressure marks in the insertion tube	Replaced the components of the insertion section	Level A	8142
Video image displayed improperly, but occasionally it displayed properly after shaking the light guide tube	Replaced the components of the insertion section	Level A	12,443
Abnormal internal arrangement due to the pressure marks in the insertion tube, and no video image displayed during angulation adjustment because the charged-coupled device wire was squeezed	Replaced the components of the insertion section	Level A	12,443
Video image displayed improperly during angulation adjustment	Replaced the components of the insertion section	Level A	12,443
Video image displayed improperly during angulation adjustment, pressure marks in the insertion tube, and water leaks in the forceps channel	Replaced the components of the insertion section	Level A	12,443
No video image during angulation, deformation and pressure marks in insertion tube, and water leaks in the forceps channel	Replaced the components of the insertion section	Level A	12,443
No video image during angulation adjustment	Replaced the components of the insertion section	Level A	12,443
Error code “E216” appeared after the flexible bronchoscope was connected to the host	Replaced the components of the insertion section	Level A	12,443
Dim video image, and pressure marks in the insertion tube	Replaced the components of the insertion section	Level A	12,443
Error code “B30” appeared, and black screen	Replaced the components of the insertion section	Level A	12,443
Black screen during angulation in down direction, water leaks in the forceps channel	Replaced the components of the insertion section	Level A	12,443
Pressure marks in the front end of the bronchoscope	Replaced the front-end rubber	Level D3	0
Water leaks at the bending rubber	Replaced the rubber	Level D3	0
Air leak	Replaced the rubber on the insertion section	Level D3	0
Front end bending	Replaced the bending rubber	Level D3	0
Visible pressure marks in the insertion section	Replaced the rubber on the insertion section	Level D3	0

The 29 pediatric flexible bronchoscope failures were categorized into the case group, while 116 instances where the pediatric flexible bronchoscopes did not have failures were classified as controls. A comparative analysis was carried out on the types and sizes of the pediatric flexible bronchoscopes, the work experience of the person responsible for flexible bronchoscope reprocessing, the time when the failure occurred, the work experience of the surgeon, and the type of surgery. Details are presented in Table 2. Table 2 shows that the failure rates in fiber bronchoscopes and electronic bronchoscopes were 34.5% and 65.5%, respectively, with a statistically significant difference; the failure rates in small-diameter bronchoscopes and large-diameter bronchoscopes were 93.1% and 6.9%, respectively, with a statistically significant difference; the failure rates in the flexible bronchoscopes reprocessed by the persons with less work experience and those reprocessed by the persons with rich work experience were 75.9% and 24.1%, respectively, with a statistically significant difference; the failure rates in the 1st, 2nd, 3rd and 4th quarters were 37.9%, 13.8%, 34.5%, and 13.8%, respectively, with a statistically significant difference; and no statistically significant differences were found in the work experience of the surgeon or type of the surgery.

**Table 2:** Influence of different factors on failures in pediatric flexible bronchoscopes

Variable	Description	Case group	Control group	Chi-square value/t value	*P*
Type of flexible bronchoscope	Fiber bronchoscope	10 (34.5)	73 (62.9)	7.671	0.006
	Electronic bronchoscope	19 (65.5)	43 (37.1)		
Size of flexible bronchoscope	Small-diameter bronchoscope	27 (93.1)	81 (69.8)	6.613	0.01
	Large-diameter bronchoscope	2 (6.9)	35 (30.2)		
Work experience of the person responsible for flexible bronchoscope reprocessing	Less	22 (75.9)	53 (45.7)	8.458	0.004
	Rich	7 (24.1)	63 (54.3)		
Time when failure occurred	The 1st quarter	11 (37.9)	25 (21.6)	8.496	0.037
	The 2nd quarter	4 (13.8)	36 (31.0)		
	The 3rd quarter	10 (34.5)	24 (20.7)		
	The 4th quarter	4 (13.8)	31 (26.7)		
Work experience of the surgeon	Less	18 (62.1)	67 (57.8)	0.178	0.673
	Rich	11 (37.9)	49 (42.2)		
Type of surgery	Foreign body removal	10 (43.5)	37 (31.9)	0.792	0.715
	Bronohoalveolarlavage	17 (58.6)	74 (63.8)		
	Other	2 (6.9)	5 (4.3)		

The factors concerning the type and size of the flexible bronchoscope, work experience of the person responsible for flexible bronchoscope reprocessing, and the time when the failure occurred were put into the multivariate logistic regression equation. In comparison to the large-diameter bronchoscopes, the small-diameter bronchoscopes had a high risk of failure (Odds ratio (OR) = 5.706, 95% confidence interval (CI) 1.226–26.548, *P* = 0.026), with a statistically significant difference. Compared with the flexible bronchoscopes reprocessed by the persons possessing rich work experience, the ones reprocessed by the persons with less work experience had a high risk of failure (OR = 3.107, 95% CI 1.179–8.187, *P* = 0.022), with a statistically significant difference, as shown in Table 3.

**Table 3:** Logistic regression analysis on different factors associated with failures in pediatric flexible bronchoscopes

Variable	Description	B	B and standard deviation	Wald chi-square value	*P*	Odds ratio	95% confidence interval for odds ratio
Type of flexible bronchoscope	Fiber bronchoscopes	0.880	0.462	3.629	0.057	0.415	0.168–1.026
	Electronic bronchoscopes^*^						
Size of bronchoscope	Small-diameter bronchoscopes	-1.741	0.784	4.928	0.026	5.706	1.226–26.548
	Large-diameter bronchoscopes^*^						
Work experience of the person responsible for flexible bronchoscope reprocessing	Less	-1.134	0.494	5.259	0.022	3.107	1.179–8.187
	Rich^*^						
Time when failure occurred		-0.254	0.213	1.422	0.233	0.776	0.511–1.178

^*^Control group

## Discussion

### Causes and influencing factors of the failures

In this study, the overall failure rate in the pediatric flexible bronchoscopes was 0.678%, which is close to the research findings of Rozman et al. [11]. Tables 1 and 2 depict that the failure rate in the electronic bronchoscopes with short service life was higher when compared to the fiber bronchoscopes with longer service life. The possible reason was that the fiber bronchoscope was composed of two fiber bundles, namely the light-transmitting bundle and the image-transmitting bundle. The light-transmitting bundle is for illumination, whereas the image-transmitting bundle, which is made up of multiple optical fibers, is the core of the fiber bronchoscope, providing high flexibility. It will not break even if bends to a certain extent [12]. The charge coupled device (CCD) is, nevertheless, at the core of the electronic bronchoscope. The image is captured by the CCD imaging system, and delivered to the image processing system via wire after being transmitted to the electrical signal. After that, the site of the lesion in the patient can be observed on the high-definition monitor screen [12]. Because the CCD is located at the front end of the insertion section of the flexible electronic bronchoscope, it is susceptible to failure. The failure rate in the small-diameter bronchoscopes, as illustrated in Tables 2 and 3 was high, which is consistent with the research results of Nishizawa et al. [13]. The shorter the external diameter, the more vulnerable a flexible bronchoscope is to damage. As a result, correct cleaning and disinfection procedures must be strictly adhered to.

There are two categories for repairs: “major” and “minor”. The major repair requires disassembling the insertion as well as the control sections of the flexible bronchoscope. Replacement of components without disassembling the insertion or control sections of the flexible bronchoscope is referred to as a minor repair. The flexible bronchoscope repairs are divided into 5 levels: A, B, C, D, and E. Among the 29 failures, 20 were classified into Level A repair in accordance with the failure description in Table 1 and the feedback from the manufacturer. Level A repair necessitates the replacement of all components in the front end structure of the flexible bronchoscope, with an average repair cost was USD10507.60.

The common failures in the pediatric flexible bronchoscopes included (1) *Black dots in the video image.* The causes could have been component aging after prolonged usage, pressure marks in the insertion tube, optical fiber breakage, external force to the front end, short circuit due to water invasion, or CCD wire breakage due to kinks in the insertion tube. (2) *Improper video image display or no image during angulation adjustment.* The front end of the flexible bronchoscope bent badly, which was one of the potential causes, as well as incorrect use during diagnosis and treatment [14]. Errors in video image display may lead to failed intubation, affecting bronchoscopic foreign body removal. (3) *Pressure marks in the insertion tube, damage to the forceps channel, and water leakage.* The causes consisted of damage to the interior surface of the flexible bronchoscope while removing the foreign body or taking tissue samples for biopsy [13], improper use of a cleaning brush during the cleaning procedure [14] (for example, mismatch between cleaning brush size and inner diameter of the bronchoscope might cause scratches on the interior surface of the bronchoscope during cleaning; or the cleaning brush had become bent and deformed after being used for a long time, but the cleaning person insisted on using it to clean the bronchoscope), not putting the pediatric flexible bronchoscope into the transport bin during transportation, coiling the bronchoscope too tightly, pressed front end, and vibration or shaking during transportation. In addition, the insertion section shook violently and collided with the host when the flexible bronchoscope was hung on the host on standby. The study of Ofstead et al. [15] reveals that improper and delayed pre-processing can lead to an increase in scrub times to ensure the quality of cleaning, resulting in the surface wear of the bronchoscope. In addition, the results of Lu et al. [16] has shown that neglect of leakage detection is a significant factor associated with the reduced service life of fiberoptic bronchoscopes. (4) *Bubbles in the bending rubber during leakage.* The rubber cracked due to aging and excessive bending angle on the rubber; or the rubber is damaged by sharp article during cleaning, disinfection, storage, and transportation [14].

Table 2 presents that failures were most common in the first and third quarters, which coincided with the rotation time for the nurses in charge of cleaning. Additionally, newly employed nurses helped clean and disinfect the pediatric flexible bronchoscopes in July. According to Tables 2 and 3, nurses with less work experience were more likely to cause failures in pediatric flexible bronchoscopes because they were not familiar with the standard bronchoscope reprocessing procedures.

### Prevention measures

#### Strict adherence to reprocessing criteria of pediatric flexible bronchoscopes

**Fig. 6:**
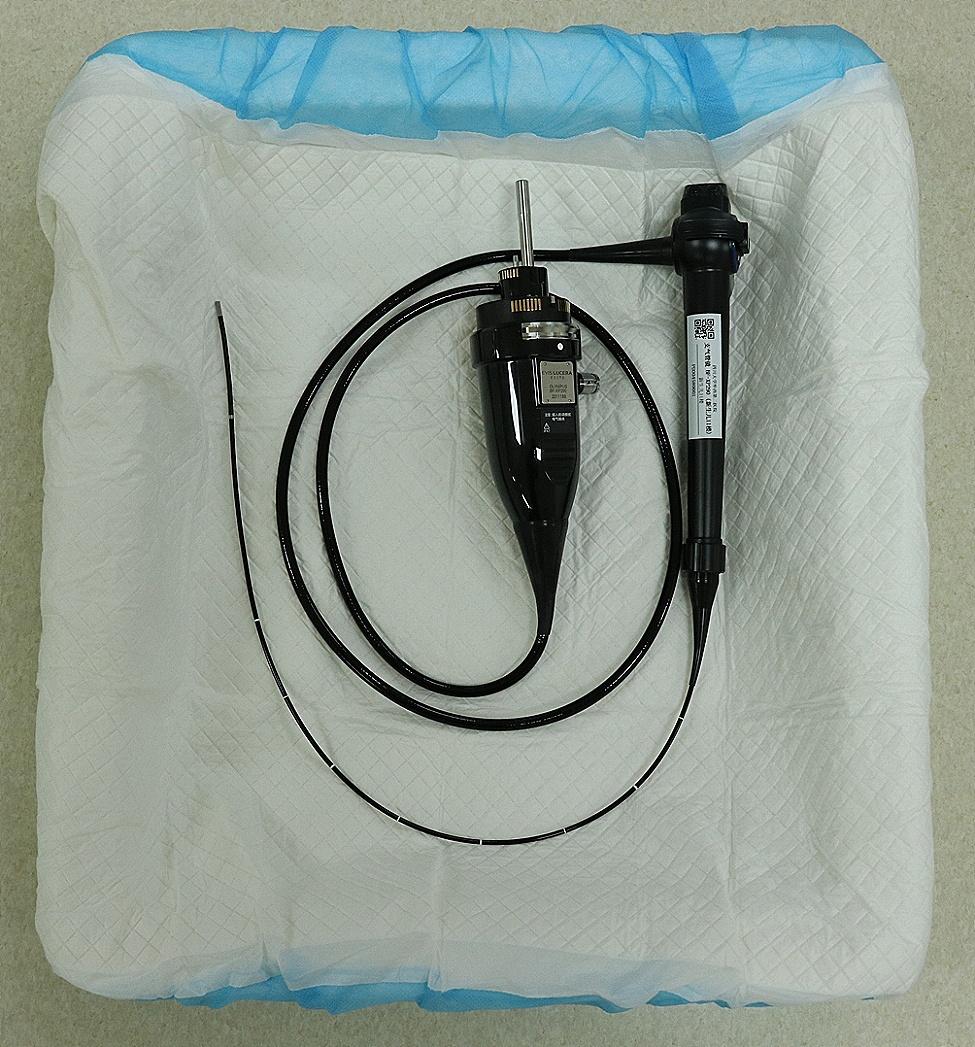
Standard coiling

**Fig. 7:**
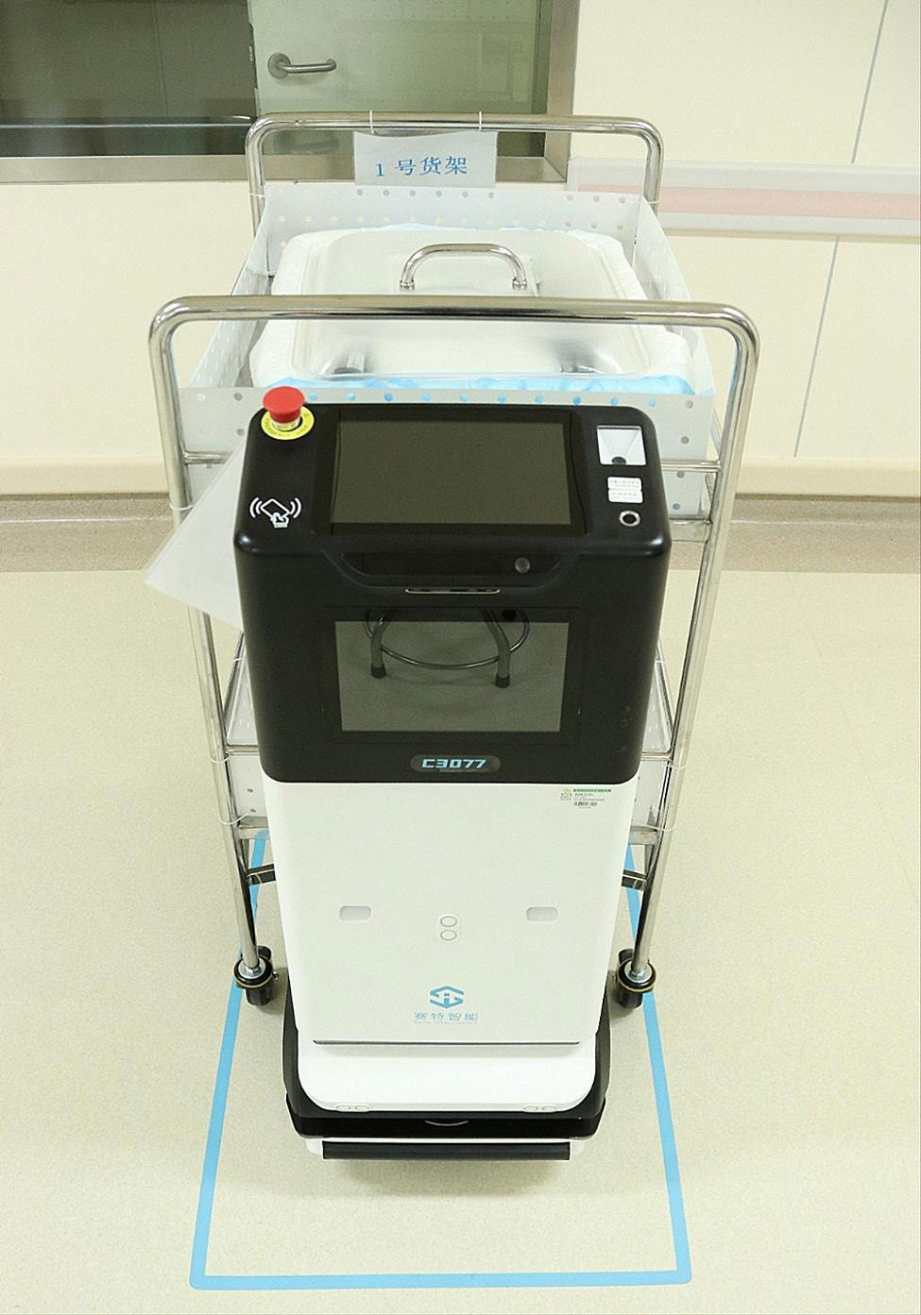
Robot for flexible bronchoscope transportation

**Fig. 8:**
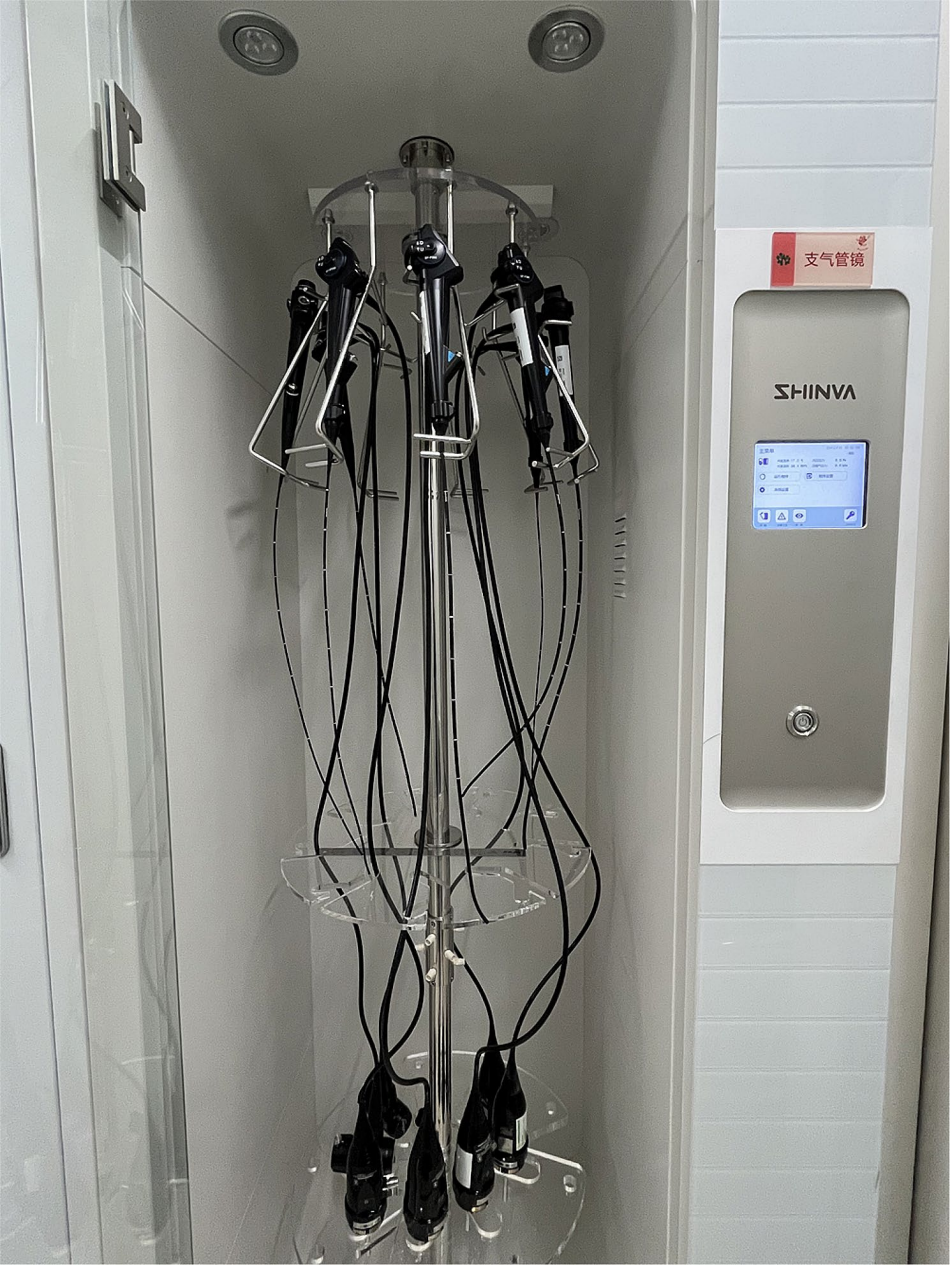
Standard storage of flexible bronchoscope

The pediatric flexible bronchoscopes shall not be squeezed or collided during collection, cleaning, drying, packaging, transportation, diagnosis and treatment [17]. Timely pre-processing and strictly following the pre-procedding procedure at the bedside is required to ensure the quality of subsequent cleaning and reduce the scrub times, thereby avoiding damage to the bronchoscopes. The bronchoscope shall be put in an upper position when being collected, cleaned, disinfected, or transported [16]. The leak test shall be performed thoroughly. The leak test is advisable to be performed prior to cleaning, and any issues should be reported right away to the technicians for assistance. Before the leak test, conducting waterproofing inspections is necessary, i.e. whether the water-resistant cap is connected to the flexible bronchoscope, and whether the components and connections are dry. The cleaning brush matching the bronchoscope specifications shall be utilized for cleaning. It is better to replace the cleaning brush in the case at the time when enormous resistance occurs during channel brushing [18]. The flexible bronchoscope shall be dried completely. The mouth of the air gun should not directly align with the channel opening for drying. For drying, it must maintain a specific distance. Sufficient space should be given in the transportation bin to avoid the damage to the bronchoscope caused by excessive coiling. The coiling shall not press the front end, and the coiling diameter must be no less than 20 cm [19]. A disposable instrument protective cover shall be put inside the transport bin for the purpose of avoiding collision between the flexible bronchoscope and the bin, as shown in Fig. 6. The stainless ruler with other articles shall be put inside the instrument protective cover so as to avoid direct contact with the flexible bronchoscope, as well as squeezing and collision during transportation [20]. It is preferable to use robots for transportation to avoid vibration and shaking during transportation by staff [21], as shown in Fig. 7. The flexible bronchoscope after drying shall be stored in the bronchoscope cabinet, and the accessories shall be placed in the accessory bin; the flexible bronchoscope should be hung vertically, with the angulation locks in the free position [22], as shown in Fig. 8.

#### Strict adherence to procedures of diagnosis and treatment

The surgeon shall strictly adhere to the following diagnosis and treatment procedures: Before taking a biopsy of tissue or removing a foreign body, check the function of the flexible bronchoscope and choose a bronchoscope in good condition for both diagnosis and treatment; a mouthpiece is placed in the patient’s mouth prior to inserting the flexible bronchoscope to prevent damage of the bending section. The surgeon shall ensure that the insertion section is not clamped, the accessory has stretched out the opening of the suction channel before locating the needle, and the biopsy forceps jaws are closed throughout both insertion and suction [21]. Besides, employing the flexible bronchoscope to meet appropriate technical requirements can likewise minimize the occurrence of failure.

#### Offering intensive training for the persons responsible for flexible bronchoscope reprocessing

Studies by Lu et al. [16] have claimed that regular training for flexible bronchoscope reprocessing handlers and strict adherence to relevant guidelines and criteria can reduce the failure rate in bronchoscopes. It is advisable to provide nurses with intensive training in relevant theories and procedures who are in charge of reprocessing pediatric flexible bronchoscopes, strengthening supervision, and performing strict assessments. Only after receiving the necessary training and passing the necessary examinations, the new hires, interns, and staff members who will switch between different positions are permitted to independently reprocess the flexible bronchoscopes. If possible, it is better to assign specific persons to reprocess the pediatric flexible bronchoscopes in order to prevent failure associated with frequent personnel changes.

### Limitations

This study has some limitations. First, this was a single-center study. A multi-center study is needed to investigate whether the results can be reproducible in other hospitals. Second, this was a retrospective study. The correlations between the investigated factors and the outcomes are exploratory. The causal relationship needs to be further confirmed by prospective studies.

## Conclusions

The Department of Sterile Processing staff shall pay increasing attention to cleaning and disinfection of pediatric flexible bronchoscopes. It is crucial to know more about the frequency of failures in pediatric flexible bronchoscopes, examine the reasons for the failures, and take the preventive measures to optimize the central workflow and management mode for reprocessing the pediatric flexible bronchoscopes. This will help to extend the service life of the flexible bronchoscopes, control repair costs, increase the clinical turnover rate, and improve clinical satisfaction.

## Data Availability

The datasets generated and/or analyzed during the current study are not publicly available due the original data is a part of the intellectual property rights, and the study also mentioned that all the collected data is kept confidential, but are available from the corresponding author on reasonable request.

## Abbreviations

CCD: Charge coupled device
OR: Odds ratio
CI: Confidence interval
